# Determinants of rural middle school students' adoption of AI chatbots for mental health

**DOI:** 10.3389/fpubh.2025.1619535

**Published:** 2025-07-17

**Authors:** Shuo Li, Lei Liu, Yuhui Wang, Xinyun Deng

**Affiliations:** School of Architecture and Art, Central South University, Changsha, China

**Keywords:** artificial intelligence, mental health, rural middle school students, UTAUT2, behavioral intention

## Abstract

Adolescent mental health challenges constitute an important global public health issue. Despite the rapid development of AI technology in various fields, its adoption in rural mental health remains constrained. The purpose of this study is to examine the factors that influence the adoption of AI chatbots for mental health education among rural Chinese secondary school students. Utilizing the UTAUT2 framework, we included Perceived Risk (PR) and Perceived Anthropomorphism (PA) to construct a theoretical model. A questionnaire survey of 317 rural adolescents was conducted, analyzed via SPSS and AMOS. Results showed PE, EE, SI, and PA positively correlated with BI; PR negatively correlated; HM had no effect. Grade level moderated specific paths. The study extends UTAUT2 to marginalized populations, filling a gap in AI-driven rural adolescent mental health interventions.

## 1 Background

Global mental health is experiencing a significant decline. According to the World Health Organization (WHO), ~1 billion people worldwide suffer from mental disorders (1), with the prevalence continuing to rise (2, 3). This increasing burden not only imposes substantial economic costs (4), but also exacerbates issues such as high unemployment (5) and low educational achievements (6), further straining the global economy (7–9). To address this challenge, the international community has implemented measures to enhance mental health (10, 11). For instance, the 2030 Sustainable Development Goals (12) advocate policy actions to promote mental wellbeing, while WHO's Global Mental Health Action Plan (13) integrates mental health services into public health systems.

Adolescents aged 10–19 are at particularly high risk of developing mental health disorders, yet their needs remain widely under-recognized and under-treated (14). Recent evidence shows that the prevalence of clinically elevated symptoms of depression and anxiety among children and adolescents reached 25.2 and 20.5% respectively during the COVID-19 pandemic, nearly double pre-pandemic levels (15).

In low-and middle-income countries, the prevalence of adolescent mental illness may be even higher (16). In China, a middle-income country, 14.8% of adolescents face mental health challenges, predominantly depression and anxiety (17). The 2022 National Depression Blue Book (18) reports that 30% of depression patients are under 18, 50% of whom are students. The 2023 China Mental Health Blue Book (19) further indicates a 40% depression detection rate among high school students and 30% among junior high students.

Adolescence is a critical period for physical and mental development. Untreated childhood mental health issues significantly increase the risks of adult health problems, criminal behavior, academic failure, and poverty (20).

Despite China's policy initiatives—such as the Special Action Plan for Student Mental Health (21–23), adolescent mental health issues remain severe (24–26). Notably, rural adolescents exhibit poorer mental health than urban peers (27). In rural schools, 20% of students show depression risk, 68% have at least one anxiety symptom (28), and their mental illness scale scores surpass urban students (29). This disparity is largely attributed to parental labor migration, which has left millions of rural students without direct parental care (30, 31).

Although cognitive behavioral therapy (CBT) (32), psychoanalytic therapy (33), pharmacological treatment (34), and complementary interventions (35–37) have proven effective in alleviating mental disorders and restoring patients' normal functioning, their implementation faces significant barriers among rural secondary school students in China. First, the exorbitant cost of professional counseling renders it financially inaccessible (38). Second, over half of the rural students reside in boarding schools (39), leaving limited time for regular counseling sessions. Furthermore, insufficient mental health literacy among parents and teachers in rural areas (40), may lead to the neglect of students' psychological issues or reluctance to seek treatment due to stigma concerns (41). In addition, many rural schools lack professional mental health teachers due to insufficient funding, limited infrastructure, and a shortage of qualified personnel (42, 43). Traditional educational priorities in rural areas emphasize academic achievement over mental health, resulting in limited recognition and support for psychological services within schools (44). Consequently, conventional mental health interventions remain impractical for most rural adolescents. This situation is similarly observed in rural and underserved communities in other countries (45–47).

In recent years, Artificial Intelligence (AI) has emerged as a transformative force, driving significant advancements in industries such as manufacturing, healthcare, architecture, and translation (48–50). As a subset of AI, chatbots are defined as intelligent programs that simulate human-like conversations (51) and have been recognized as a promising solution for mental health interventions (52). Within mental health domains, AI technologies—particularly generative AI—demonstrate capabilities in comprehending and generating natural language, with performance comparable to or exceeding human expertise in medical diagnosis, communication, and therapeutic practices (53). These AI-driven chatbots are based on robust decision-making framework. According to Guo and Hou (54) their research analyzes the conversational context to identify users' emotional states and subsequently utilize risk stratification models to assess mental health conditions and deliver personalized interventions. Unlike human therapists, AI chatbots offer round-the-clock support (55), providing a cost-effective solution to address mental health resource shortages in rural areas and potentially enhancing students' psychological wellbeing.

In summary, AI holds significant promise for delivering mental health education to rural secondary school students. However, existing research predominantly focuses on AI applications in universities (56) and urban primary/secondary schools (57), while targeted studies on rural adolescents remain scarce. Given the low adoption rates of AI technologies in rural settings, students' acceptance of such systems may significantly influence intervention efficacy. This study therefore aims to investigate rural students' acceptance of AI chatbots and its determinants, addressing current research gaps and proposing a scalable intervention framework for global implementation.

Part 1 reviews global mental health challenges and examines mental health issues prevalent among secondary school students in Chinese educational contexts. Part 2 examines key studies from existing literature, demonstrating the feasibility of AI applications in mental health education. Part 3 details the questionnaire-driven methodology and UTAUT2-derived research model. Parts 4–5 detail the survey implementation, data analysis procedures, and empirical findings. Part 6 discusses the implications of these findings and proposes evidence-based recommendations. The paper concludes with a synthesis of major contributions and future directions.

## 2 Related research

### 2.1 Application of artificial intelligence in educational contexts

Recent studies indicate that artificial intelligence (AI) offers transformative opportunities for the education sector, with applications extensively penetrating core domains such as curriculum development, learning guidance, and instructional assistance.

First, research on curriculum development—the cornerstone of educational practice—has yielded AI-powered frameworks and tools to enhance pedagogical design. For instance, Dickey and Bejarano (58) proposed the GAIDE framework, enabling educators to efficiently develop diverse, high-quality, and learner-centric materials through AI, thereby alleviating workload pressures. Heo and Kang (59) introduced the AIESTEP platform, which integrates learning content with practical modules and facilitates real-time teacher reflection for curriculum optimization. Sun et al. (60) further confirmed AI's efficacy in improving teachers' technical proficiency, self-efficacy, and instructional outcomes through experimental evidence.

The second research direction focuses on personalized learning guidance. In educational environments, students exhibit diverse learning experiences, proficiency levels, and individual characteristics. To enhance both engagement and knowledge retention, AI technology can dynamically adjust instructional content and methodologies based on learners' behavioral patterns, cognitive styles, and learning preferences (61), delivering tailored educational experiences (62). Representative implementations include Becerra et al. (63), who utilized generative AI to analyze MOOC data for personalized interventions and dropout prevention, and Qiu et al. (64), who developed the “Academic Quick Guide” system to streamline educational administration through 24/7 academic support.

The third direction emphasizes AI-augmented pedagogical support. As interactive practice constitutes a crucial component for assessing knowledge acquisition, some researchers have conducted related studies on this recently. Lin and Ye (65) developed a biology-focused chatbot to enhance academic performance via extracurricular scaffolding; Xu et al. (66) implemented a digital game-based chatbot system that boosts motivation through gameplay mechanics; while Banjade et al. (67) created an adaptive learning environment integrating AI-generated images with text-to-speech technology.

Notably, AI demonstrates particular efficacy in addressing educational disparities within China's resource-constrained rural regions. Niu et al. (68) mixed-methods study involving 130 teachers and students across nine schools, validated the effectiveness of AI platforms as effective pedagogical tools, particularly in resource-limited contexts.

### 2.2 Application of artificial intelligence in psychotherapy

Artificial intelligence has been explored in the mental health domain for decades. Pioneering systems include Eliza, which simulates psychotherapeutic dialogues to investigate problem origins (69). Woebot implements cognitive behavioral therapy to detect and mitigate depression (70), and Tess delivers emotional support to reduce anxiety and depression levels (71). Additionally, iHelpr facilitates depressive symptom self-assessment with improvement recommendations (72). Contemporary AI applications exhibit enhanced specialization and comprehensiveness in psychotherapy.

The primary consideration lies in supplementing or replacing traditional treatment methods. M and Nallasamy (73) demonstrate that chatbots delivering behavioral therapy through virtual coaching can enhance clinical outcomes, reduce social stigma, and bridge treatment gaps. Vahedifard et al. (74) analyze ChatGPT's potential in psychiatry, acknowledging its emotional support capabilities while examining privacy and ethical concerns. Eid et al. (75) leverage AI-powered patient data analysis to optimize depression treatment plans to enable precision medication customization.

Special population factors constitute another critical dimension. Habicht et al. (76) reveal through studies on marginalized groups (e.g., bisexual individuals) that AI chatbots significantly lower treatment barriers compared to conventional approaches, promoting equitable access to mental health services. Wang and Li (77) establish through comparative experiments that AI interventions not only alleviate geriatric depression but also reduce economic burdens.

Regarding therapeutic efficacy for student mental health, Mahmud and Porntrakoon (78) propose AI as a viable complement or alternative to traditional treatments for Thai university students, though AI requires enhanced user-friendly designs and privacy safeguards. Moreover, Oghenekaro and Okoro (79) validate through combined quantitative-qualitative assessments that AI technologies provide personalized support significantly improving psychological states. Klos et al. (80) confirmed Tess's (https://tess.x2ai.com/) intervention potential for anxiety and depression in Argentine university students via controlled trials. Liu et al. (81) demonstrated that chatbot-based self-help interventions outperformed bibliotherapy in depression management, and Wang et al. (82) documented high trust levels of middle school students in using AI wristbands for mental health intervention.

Notably, current research predominantly focuses on hospital patients and university populations (83). Research on middle school students, particularly in rural areas, remain critically underexplored.

### 2.3 Unified theory of acceptance and use of technology (UTAUT) in AI applications for mental health

The UTAUT model provides robust theoretical foundation for investigating users‘ acceptance and adoption behaviors toward emerging technologies. Through its core constructs of Performance Expectancy, Effort Expectancy, and Social Influence, this framework systematically analyzes key drivers underlying rural secondary school students' utilization of AI-powered psychotherapeutic tools. The model enables systematic understanding and prediction of this population's acceptance of AI mental health interventions, offering theoretical guidance for future implementation designs. Several scholars have employed UTAUT to explore AI applications in mental health contexts. For instance, Alojail (84) utilized UTAUT to examine user acceptance of digital mental health interventions, revealing that perceived usefulness, perceived ease of use, and facilitating conditions exerted positive effects on user attitudes and behavioral intentions, whereas social influence demonstrated non-significant effects. Henkel et al. (85) extended UTAUT to LGBTQIA+ populations in mental health chatbot adoption studies, identifying Performance Expectancy, Social Influence, Willingness to Self-disclose, and Trust as critical determinants of behavioral intention, with gender showing no moderating effects. Li et al. (136) investigated Americans with depression/anxiety, finding Performance Expectancy, Price Value, and Descriptive Norms were all positively related to behavioral intention for both treatment-naïve and experienced cohorts, while Effort Expectancy was negatively associated with behavioral intention.

These findings suggest AI adoption intentions correlate closely with perceived usefulness, perceived ease of use, and risk perceptions. Therefore, applying the UTAUT framework to investigate influencing factors in rural adolescents' acceptance of AI-assisted mental health education proves methodologically appropriate.

## 3 Research methods

### 3.1 Theoretical model

The UTAUT is an Integrated Technology Acceptance Model proposed by Venkatesh et al. (86) through synthesizing and refining prior Technology Acceptance Model (TAM) research. Venkatesh et al. (87) further refined and validated the UTAUT framework, introducing the UTAUT2 model. This enhanced framework incorporates additional variables to comprehensively explain and predict users' acceptance and adoption behaviors toward emerging technologies. The UTAUT2 model operationalizes seven core constructs including Performance Expectancy, Effort Expectancy, Social Influence, Hedonic Motivation, Price Value, Facilitating Conditions, and Habit, along with three moderators which are Gender, Age, and Experience. Empirical validation demonstrates that this model explains 74% of the variance in behavioral intention across diverse cultural and social contexts, significantly outperforming the original UTAUT framework (88). Consequently, UTAUT2 serves as the theoretical foundation for this study.

### 3.2 Variable selection and hypotheses

#### 3.2.1 Variable selection

**Figure 1:**
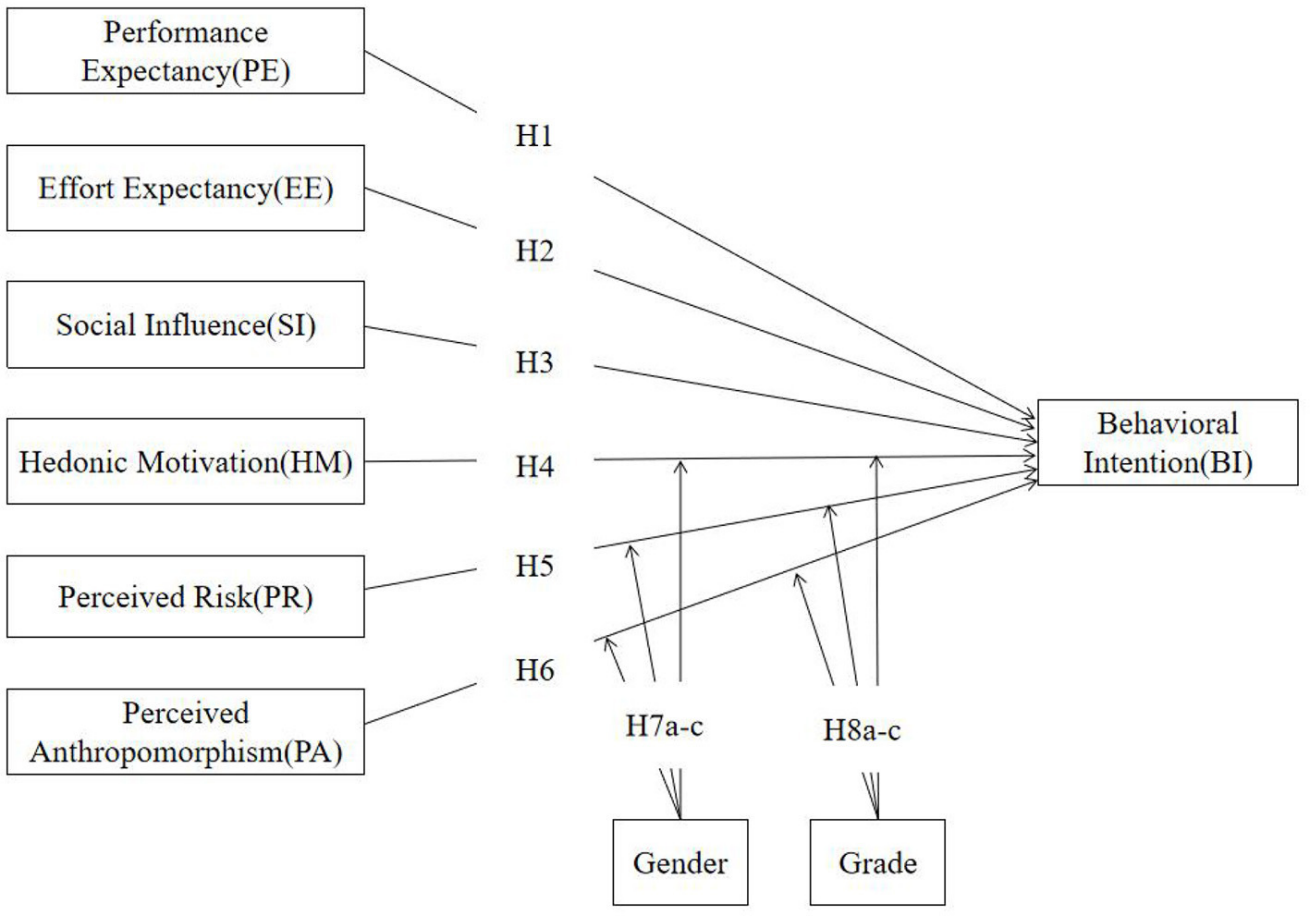
Research model for measuring the adoption of AI chatbots.

This study retains four core constructs from the original UTAUT2 model: Performance Expectancy (PE), Effort Expectancy (EE), Social Influence (SI), and Hedonic Motivation (HM). Facilitating Conditions (FC) and Price Value (PV) were excluded because education departments will provide schools with equipment and training for free to facilitate students' use. Given the exploratory nature of AI-assisted mental health education in rural Chinese secondary schools, we removed habit and experience variables from UTAUT2. This decision was also influenced by students' limited experience and developing autonomy. However, we retained gender as a moderator. The age variable has been replaced by the grade moderating variable to examine behavioral intention variations across different academic stages. Considering that students in rural Chinese secondary schools have less exposure to AI technology, they may be concerned about privacy risks when using such unfamiliar tools. Moreover, as mental health education involves emotional interactions, human-like chatbots may affect students' attitudes toward usage. Therefore, Perceived Risk (PR) and Perceived Anthropomorphism (PA) are included as variables. Our research model is shown in Figure 1.

#### 3.2.2 Hypotheses development

Performance expectancy is defined as the degree to which an individual believes using the system will enhance job performance (89). Huang et al. (90) confirmed in their research on health chatbots that performance expectancy is a crucial variable. As a well-established predictor of behavioral intention (86), performance expectancy in this context reflects rural adolescents' perceptions that AI chatbots can improve mental health outcomes. Students who perceive AI as effective in addressing psychological issues or enhancing wellbeing are more likely to demonstrate higher behavioral intention. We derive the following hypothesis from this:

**H1**. PE positively influences rural students' BI toward AI-assisted mental health education.

Effort expectancy is defined as the perceived ease or difficulty of utilizing a technology (87). Prior studies have shown that effort expectancy plays a vital role in the use of Learning Management System (LMS) and AI Tools (91, 92). Specifically, effort expectancy in this study measures the effort and time investment required for rural students to achieve desired psychological improvements through AI chatbot interactions. If students perceive the AI system as user-friendly, easy to understand and demanding minimal learning effort, their willingness to adopt the technology will markedly increase. Thus, the following hypothesis was proposed:

**H2**. EE positively influences rural students' BI toward AI-assisted mental health education.

Social influence refers to the extent to which an individual is influenced by the behavior of groups from outside (87). Nurkhin (93) confirmed that social influence has a positive impact on the willingness to use online learning. In this study, social influence refers to whether rural secondary school students are influenced by their peers, parents, and teachers when using AI chatbots. If students' peers or people around them widely recognize and actively use AI chatbots, students themselves may also be more inclined to adopt them. Therefore, the following hypothesis was proposed:

**H3**. SI positively influences rural students' BI toward AI-assisted mental health education.

Hedonic motivation denotes the fun and pleasure consumers feel when using a technology (87). Earlier investigations have found that hedonic motivation has the strongest influence on the behavioral intention to use mobile applications (94, 95). In this study, hedonic motivation refers to whether or not rural secondary school students feel pleasure when using an AI chatbot. If students find that using the chatbot leads to a pleasurable experience, it may increase their willingness to continue using it. We deduce the following hypothesis:

**H4**. HM positively influences rural students' BI toward AI-assisted mental health education.

Perceived risk refers to users' uncertainty regarding potential risks associated with technology adoption (87). For example, concerns about data privacy vulnerabilities (96). Empirical evidence indicates a negative correlation between perceived privacy loss and chatbot acceptance (97). Wu et al. (98) substantiated that perceived risk (PR) significantly negatively impacts students' AI-assisted learning. In this study, students' behavioral intentions may diminish if they perceive AI technologies as posing privacy threats. Thus, we propose:

**H5**. PR negatively influences rural students' BI toward AI-assisted mental health education.

Perceived anthropomorphism in AI chatbots refers to endowing machines with human-like traits, thereby enabling natural language conversational capabilities (99). For users, the presence of empathy and empathic abilities in therapeutic chatbots is positively correlated with their behavioral intention (100). In this study, the degree of anthropomorphism influences students' trust and emotional affinity toward AI chatbots. If a chatbot exhibits higher anthropomorphic features, students may show greater willingness to interact with it, thereby increasing their adoption intention. This leads to the following hypothesis:

**H6**. PA positively influences rural students' BI toward AI-assisted mental health education.

Gender and age are recognized moderators of technology acceptance (101). In this study, age is operationalized as grade level, with both gender and grade hypothesized to shape attitudes toward chatbots. Hence, the hypotheses:

**H7**. Gender moderates the effects of HM (H7a), PR (H7b), and PA (H7c) on BI.
**H8**. Grade moderates the effects of HM (H8a), PR (H8b), and PA (H8c) on BI.

## 4 Scale design and data collection

This article designed scales based on the characteristics of each variable and collected data through the distribution of questionnaires.

### 4.1 Questionnaire design

Based on the above hypotheses and theoretical framework, this study developed a survey instrument by adapting validated measurement scales from prior literature. The constructs of performance expectancy, effort expectancy, social influence, hedonic motivation, and behavioral intention were drawn directly from the Unified Theory of Acceptance and Use of Technology 2 (UTAUT2) proposed by Venkatesh et al. (87). The original items were slightly reworded to fit the context of AI chatbots for mental health—for example, by replacing general terms such as “technology” or “system” with “AI chatbot.” These subscales have been widely validated and applied in studies involving both general consumers and student populations. The perceived risk scale was adapted from Liu and Tao (102), originally developed in the context of smart healthcare services, while the perceived anthropomorphism scale was adapted from Liu and Cao (103), who examined users' responses to human-like virtual chatbots. In both cases, domain-specific terms (e.g., “smart healthcare services” or “Alex/Robo”) were replaced with references to AI chatbots, while preserving the original constructs. All items were further reviewed to ensure clarity, contextual relevance, and age appropriateness for secondary school students.

The questionnaire comprises two primary parts. Firstly, it includes demographic information, namely gender and grade level. Secondly, it contains influencing factor metrics. These metrics are structured across seven dimensions, and there are a total of 21 measurement items in this section. For detailed information about the instrument and source references, please refer to Table 1. All items were quantified using a 5-point Likert scale, where 1 indicates “strongly disagree” and 5 denotes “strongly agree.” Prior to formal data collection, a pilot test was conducted with 65 respondents to assess the questionnaire's reliability and validity. The instrument was subsequently refined based on pilot test outcomes to establish the final version for official administration. Table 1 displays the finalized items.

**Table 1:** Measurement items and corresponding references.

**Construct**	**Item**	**Measurement**	**Reference**
Performance expectancy	PE1	Using such an AI chatbot will benefit my mental health	(87)
	PE2	Using such an AI chatbot will improve my mental health	
	PE3	Using such an AI chatbot will improve my mental health faster	
Effort expectancy	EE1	I find it easy to learn how to use such an AI chatbot	(87)
	EE2	The operation of such an AI chatbot is clear and easy for me to understand	
	EE3	I believe I could master using such an AI chatbot without external help.	
Social influence	SI1	My friends and family think I should use such an AI chatbot.	(87)
	SI2	People around me will influence me to use such an AI chatbot.	
	SI3	My campus environment supports my use of such an AI chatbot	
Hedonic motivation	HM1	It is fun to use such an AI chatbot.	(87)
	HM2	Using such an AI chatbot is an enjoyable experience	
	HM3	Using such an AI chatbot is fulfilling	
Perceived risk	PR1	I am worried that using such an AI chatbot will collect too much personal information from me	(102)
	PR2	I am worried that such an AI chatbot will use my personal information for other purposes without my authorization.	
	PR3	I am concerned that such an AI chatbot will share my personal information with other entities without my authorization	
Perceived anthropomorphism	PA1	Communicating with such an AI chatbot is like engaging with a real human being	(103)
	PA2	Such an AI chatbot is able to socialize like a human and has its own consciousness	
	PA3	Communicating with an AI chatbot like this makes me feel warm.	
Behavioral intention	BI1	I plan to use such an AI chatbot in the future.	(87)
	BI2	I plan to use this AI chatbot frequently.	
	BI3	I would recommend this AI chatbot to others.	

### 4.2 Data collection

China has established the world's largest education system (104). Henan Province, which has the largest basic education population nationwide (105), represents a typical educational demographic. For instance, Zhoukou City contains a rural population exceeding 50% of its total residents with 676,900 students enrolled in general secondary schools (106, 107). These statistical profiles effectively reflect shared characteristics of educational contexts in China's rural areas. Consequently, Zhoukou was selected as the case study area to investigate rural middle school students' behavioral intentions toward AI chatbots through questionnaire surveys.

The formal survey via the Questionnaire Star platform (https://www.wjx.cn/) consisted of two phases. In Phase I, questionnaires were electronically distributed via WeChat parent-teacher groups, yielding 202 responses (all participants from Zhoukou rural schools). Before answering the questionnaire, all participants were required to watch an embedded instructional video introducing the concept, functions, and limitations of AI chatbots for mental health support. This ensured that participants had a consistent and objective understanding of the chatbot. After excluding invalid responses (e.g., short-duration submissions and uniform answers), 187 valid questionnaires were retained. In Phase II, a stratified random sampling method was employed (25 students per grade, none of whom participated in the first survey) to conduct an in-person survey during noon study sessions. Participants first viewed an AI chatbot instructional video, followed by onsite questionnaire completion under researcher supervision. From 150 collected responses, 130 met the validity criteria after data cleansing.

This study was conducted ethically. All participants signed an informed consent form prior to the survey. Participation was voluntary and anonymous. Since the AI chatbot was presented only in a simulated video, no real interaction or psychological risk was involved.

As shown in Table 2, the sample comprised 185 male participants (58.36%) and 132 female participants (41.64%). Regarding academic progression, 51.42% were junior high school students, while 48.58% were senior high school students.

**Table 2:** Demographic characteristics of participants (*n* = 317).

**Variable**	**Level**	**Count**	**Proportion (%)**
Gender	Male	185	58.36
	Female	132	41.64
Grade	7	42	13.25
	8	63	19.87
	9	58	18.30
	10	70	22.08
	11	50	15.77
	12	34	10.73

## 5 Data analysis

This study employed SPSS 26 and AMOS 23 for data processing and hypothesis testing. The analytical procedure consisted of five stages. First, reliability analysis of the scales was conducted using SPSS. Subsequently, confirmatory factor analysis (CFA) was performed in AMOS to assess structural validity, and exploratory factor analysis (EFA) in SPSS was employed concurrently to examine dimensionality and discriminant validity. Next, descriptive statistics and normality tests were conducted through SPSS. Then, structural equation modeling (SEM) was constructed via AMOS. Finally, hierarchical regression analysis was implemented to evaluate the impacts of demographic variables on research outcomes.

### 5.1 Reliability analysis

To ensure measurement quality, scale reliability was initially verified as a prerequisite for subsequent analyses. Internal consistency across dimensions was evaluated using Cronbach's alpha coefficient (α), where values range from 0 to 1 with higher coefficients indicating superior reliability. The established thresholds were: < 0.6 (unacceptable), 0.6–0.7 (acceptable), 0.7–0.8 (good), 0.8–0.9 (excellent) and >0.9 (ideal). Using 317 completed questionnaires, the calculated α values for all seven latent variables exceeded 0.7 (Table 3). These results confirm the measurement scale demonstrates satisfactory internal consistency and meets psychometric standards.

**Table 3:** Reliability analysis using Cronbach's α.

**Construct**	**Cronbach's α**	**Composite Cronbach's α**
Performance expectancy	0.768	0.817
Effort expectancy	0.779	
Social influence	0.809	
Hedonic motivation	0.780	
Perceived risk	0.834	
Perceived anthropomorphism	0.768	
Behavioral intention	0.798	

### 5.2 Validity test

**Figure 2:**
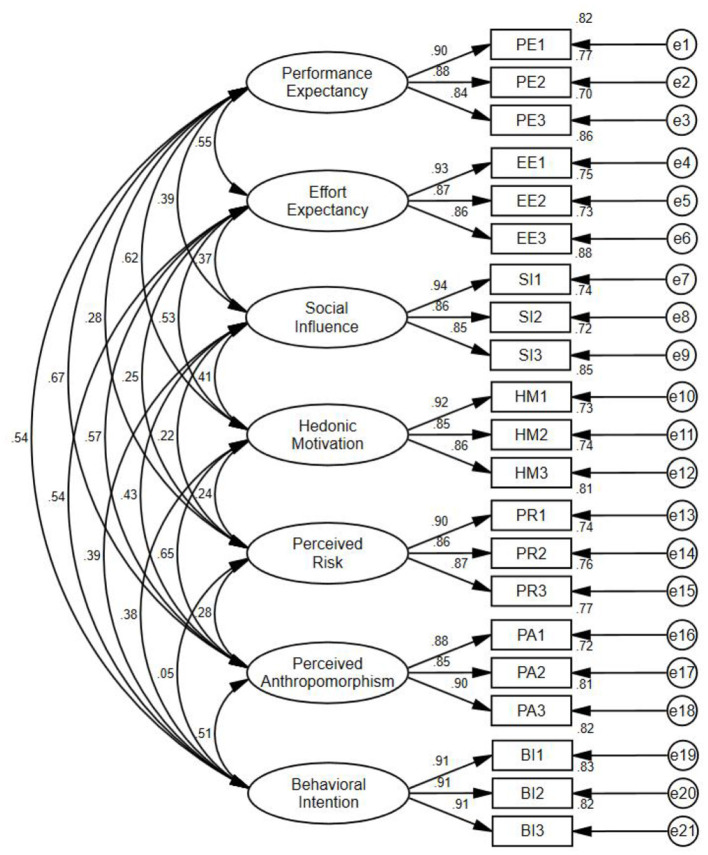
Confirmatory factor analysis results.

Validity tests were conducted to verify whether the observed variables accurately reflect the latent variables, ensuring the validity of the questionnaire. Since all observed variables in this questionnaire were derived from previously validated scales, confirmatory factor analysis (CFA) was employed to examine the alignment between factor-observed variable relationships and theoretical assumptions. In this study, confirmatory factor analysis (CFA) was conducted using AMOS software (IBM SPSS, Chicago, OH, USA), with the analytical results illustrated in Figure 2. As shown in Table 4, the model fit indices demonstrated satisfactory performance: the CMIN/DF (chi-square/degrees of freedom ratio) of 2.502 fell within the recommended range of 1–3, and the RMSEA (root mean square error of approximation) of 0.059 remained below the 0.08 threshold, indicating acceptable model fit. Furthermore, other indices including the NFI (normed fit index) and CFI (comparative fit index) achieved good levels exceeding 0.9. These results confirm the model's strong fit, validating the questionnaire's effectiveness.

**Table 4:** Model fit indices and criteria.

**Indicator**	**Judgment criteria**		**Suitability**	**Test results**
	**Acceptable**	**Good**		
CMIN/DF	3–5	1–3	2.502	Good
GFI	>0.8	>0.9	0.889	Acceptable
CFI	>0.8	>0.9	0.955	Good
NFI	>0.8	>0.9	0.927	Good
RMSEA	< 0.08	< 0.05	0.059	Acceptable

CFA encompasses convergent validity and discriminant validity. Convergent validity was assessed via composite reliability (CR) and average variance extracted (AVE). The AVE value, a critical metric for evaluating convergent validity, determines the strength of relationships between measurement items and their corresponding factors. Higher AVE values indicate greater reliability and convergent validity of the construct, with an ideal threshold exceeding 0.5. Composite reliability (CR) reflects the internal consistency of observed variables, where higher CR values signify stronger internal consistency and convergence. As shown in Table 5, all seven latent variables exhibited CR values above 0.7 and AVE values exceeding 0.5, confirming satisfactory composite reliability and convergent validity. Additionally, all standardized factor loadings surpassed 0.7, indicating strong explanatory power of individual items for their respective dimensions. Collectively, these results demonstrate robust convergent validity and composite reliability across all dimensions.

**Table 5:** Convergent validity and construct reliability.

**Construct**	**Item**	**Factor loading**	**AVE**	**CR**
Performance expectancy	PE1	0.877	0.762	0.906
	PE2	0.838		
	PE3	0.927		
Effort Expectancy	EE1	0.866	0.781	0.914
	EE2	0.857		
	EE3	0.938		
Social influence	SI1	0.858	0.778	0.913
	SI2	0.848		
	SI3	0.920		
Hedonic motivation	HM1	0.854	0.771	0.910
	HM2	0.859		
	HM3	0.902		
Perceived risk	PR1	0.861	0.773	0.911
	PR2	0.873		
	PR3	0.878		
Perceived anthropomorphism	PA1	0.849	0.767	0.928
	PA2	0.901		
	PA3	0.906		
Behavioral intention	BI1	0.909	0.822	0.933
	BI2	0.905		
	BI3	0.877		

The data were subjected to varimax rotation to elucidate factor-item correlations. Given the seven predefined variables in the theoretical model, principal component analysis extracted seven common factors, yielding the rotated component matrix in Table 6. Analysis of factor loading coefficients (highlighted in bold) revealed communality values exceeding 0.4 for all items, indicating strong item-factor associations and effective information extraction by the factors. Furthermore, the grouping patterns of measurement indicators under each variable aligned with theoretical expectations.

**Table 6:** Factor loadings after varimax rotation.

**Item**	**Factor loading**						
	**Factor 1**	**Factor 2**	**Factor 3**	**Factor 4**	**Factor 5**	**Factor 6**	**Factor 7**
PE1	0.198	0.077	0.116	0.284	0.162	**0.812**	0.220
PE2	0.221	0.136	0.036	0.182	0.185	**0.818**	0.235
PE3	0.193	0.185	0.185	0.213	0.212	**0.752**	0.211
EE1	0.256	0.128	0.035	0.179	**0.853**	0.132	0.191
EE2	0.252	0.088	0.123	0.170	**0.819**	0.138	0.184
EE3	0.107	0.138	0.165	0.185	**0.823**	0.250	0.145
SI1	0.166	**0.878**	0.077	0.153	0.110	0.113	0.166
SI2	0.114	**0.901**	0.061	0.104	0.111	0.042	0.049
SI3	0.100	**0.868**	0.087	0.118	0.081	0.164	0.137
HM1	0.082	0.160	0.129	**0.840**	0.210	0.189	0.213
HM2	0.096	0.125	0.027	**0.820**	0.181	0.212	0.229
HM3	0.161	0.143	0.072	**0.830**	0.134	0.198	0.206
PR1	0.038	0.124	**0.902**	0.068	0.107	0.102	0.072
PR2	−0.045	−0.004	**0.905**	0.085	0.068	0.067	0.080
PR3	−0.018	0.090	**0.910**	0.031	0.075	0.065	0.073
PA1	0.161	0.086	0.110	0.248	0.180	0.244	**0.809**
PA2	0.123	0.212	0.084	0.244	0.217	0.227	**0.780**
PA3	0.269	0.148	0.122	0.246	0.175	0.203	**0.790**
BI1	**0.859**	0.167	−0.001	0.067	0.157	0.253	0.153
BI2	**0.877**	0.110	−0.042	0.123	0.184	0.182	0.142
BI3	**0.879**	0.137	0.001	0.133	0.218	0.097	0.161

Bold values indicate the highest factor loading for each item, representing its primary association with a specific component after varimax rotation.

Discriminant validity, which ensures the distinct measurement of different constructs, was evaluated by comparing the square roots of AVE values with inter-factor correlation coefficients. As presented in Table 7, all AVE square roots exceeded the absolute values of corresponding inter-factor correlations, confirming adequate discriminant validity of the measurement model.

**Table 7:** Discriminant validity test results.

**Construct**	**PE**	**EE**	**SI**	**HM**	**PR**	**PA**	**BI**
PE	**0.873**						
EE	0.520	**0.884**					
SI	0.365	0.333	**0.882**				
HM	0.566	0.487	0.368	**0.878**			
PR	0.256	0.245	0.189	0.204	**0.879**		
PA	0.611	0.525	0.388	0.596	0.250	**0.876**	
BI	0.496	0.492	0.345	0.357	0.041	0.467	**0.907**

The values on the diagonal (in bold) are the square root of average variance extracted (AVE) estimates.

### 5.3 Descriptive statistics and normality test

Table 8 presents descriptive statistics and normality test results for the measured constructs in this study. The analysis of descriptive statistics reveals that all variables' mean scores range between 3 and 4. Given the 1–5 positive scoring scale, these results indicate that participants' awareness of using AI chatbots for mental health education assistance is above medium level. Normality tests for measurement items were conducted through skewness and kurtosis analyses. Following Kline's (137) criteria (absolute skewness < 3; absolute kurtosis < 8), the data were considered approximately normally distributed. As evidenced in Table 8, all measurement items' absolute skewness and kurtosis values fall within these thresholds, confirming their compliance with normal distribution assumptions.

**Table 8:** Descriptive statistics and normality test results.

**Construct**	**Item**	**Mean scores**	**Standard deviation**	**Kurtosis**	**Skewness**
PE	PE1	3.669	1.106	−0.786	−0.004
	PE2	3.650	1.139	−0.639	−0.403
	PE3	3.653	1.093	−0.651	−0.251
EE	EE1	3.804	1.079	−0.897	0.299
	EE2	3.817	1.045	−0.681	−0.309
	EE3	3.842	0.997	−0.759	0.124
SI	SI1	3.221	1.148	−0.403	−0.693
	SI2	3.350	1.161	−0.419	−0.724
	SI3	3.262	1.155	−0.301	−0.741
HM	HM1	3.703	1.013	−0.611	−0.153
	HM2	3.675	1.144	−0.781	−0.082
	HM3	3.625	1.035	−0.734	0.166
PR	PR1	3.845	0.874	−0.896	1.094
	PR2	3.804	0.941	−0.930	0.873
	PR3	3.845	0.927	−0.912	0.919
PA	PA1	3.580	1.027	−0.765	0.205
	PA2	3.634	0.999	−0.651	0.012
	PA3	3.612	1.051	−0.784	0.051
BI	BI1	3.438	1.088	−0.568	−0.325
	BI2	3.385	1.184	−0.437	−0.693
	BI3	3.404	1.151	−0.422	−0.677

### 5.4 Structural equation analysis

As shown in Table 9, PE (β = 0.261, *p* < 0.001), EE (β = 0.281, *p* < 0.001), SI (β = 0.136, *p* < 0.01), and PA (β = 0.176, *p* < 0.01) exerted significant positive effects on BI. Thus, H1, H2, H3 and H6 were supported. PR (β = −0.154, *p* < 0.01) demonstrated a negative association with BI, validating H5. Contrary to expectations, HM (β = −0.052, *p* > 0.10) showed no significant relationship with BI, leading to the rejection of H4.

**Table 9:** Standardized path coefficients of the model.

**Path**			**Unstandardized estimation**	**S.E**.	**C.R**.	***p***	**β**	***f* ^2^**	**Results**
PE	→	BI	0.275	0.065	4.252	0.000	0.261	0.326	Supported
EE	→	BI	0.313	0.063	5.005	0.000	0.281	0.319	Supported
SI	→	BI	0.137	0.050	2.726	0.006	0.136	0.135	Supported
HM	→	BI	−0.057	0.065	−0.864	0.387	−0.052	-	No effect
PR	→	BI	−0.196	0.059	−3.294	0.001	−0.154	0.002	Negatively correlated
PA	→	BI	0.200	0.072	2.791	0.005	0.176	0.279	Supported

The effect size for each predictor was evaluated using Cohen's *f*^2^, which indicates the unique contribution of each variable to the explained variance in BI. According to Cohen's (108) benchmarks, *f*^2^ values of 0.02, 0.15, and 0.35 represent small, medium, and large effects respectively. In this study, PE, EE, and PA showed medium to large effect sizes, SI and PR exhibited a small effect size.

### 5.5 Effect of moderating variables

The path analysis results indicate that the hypothesized relationship between HM and BI (H4) was not supported. Consequently, hypotheses H7a and H8a were rejected, and their moderation effects were not further examined.

This study employed hierarchical regression analysis to investigate the moderating effects of gender and grade. The analytical framework positioned PR and PA as independent variables, and BI as the dependent variable, with gender and grade as moderators. Interaction terms were systematically incorporated into sequential regression models.

As shown in Table 10, with gender included in Model 2, neither the PR × Gender (β = 0.008, *p* > 0.05) nor PA × Gender (β = −0.03, *p* > 0.05) interaction terms reached statistical significance. These results confirm that there are no gender moderation effects on the relationship between PR and BI. Similarly, there are no such effects on the relationship between PA and BI. As a result, hypotheses H7b and H7c are rejected.

**Table 10:** Gender moderation effects analyzed using hierarchical regression.

**PR**	**Dependent variable: BI**		**PA**	**Dependent variable: BI**	
	**Model 1**	**Model 2**		**Model 3**	**Model 4**
PR	0.047	0.041	PA	0.466^**^	0.466^**^
Gender	0.044	0.044	Gender	0.022	0.023
PR × Gender	/	0.008	PA × Gender	/	−0.03
*R* ^2^	0.004	0.004	*R* ^2^	0.219	0.22
Δ*R*^2^	0.002	0.000	Δ*R*^2^	0	0.001
F	0.566	0.379	F	43.936^**^	29.355^**^

^*^p < 0.05,

** *p* < 0.01.

As shown in Table 11, after incorporating grade into the model, the interaction term PR × Grade showed a significantly positive coefficient (β = 0.132, *p* < 0.05), indicating that grade exerts a significant positive moderating effect on the relationship between PR and BI, thus supporting Hypothesis H8b. This moderation effect corresponds to a Δ*R*^2^ of 0.017, indicating a moderate level of effect size. In contrast, the PA × Grade interaction term had no significant effect on BI (β = −0.014, *p* > 0.05), demonstrating that the moderator grade does not significantly influence the relationship between PA and BI, leading to the rejection of Hypothesis H8c.

**Table 11:** Grade moderation effects analyzed using hierarchical regression.

**PR**	**Dependent variable: BI**		**PA**	**Dependent variable: BI**	
	**Model 1**	**Model 2**		**Model 3**	**Model 4**
PR	0.027	0.031	PA	0.460^**^	0.459^**^
Grade	0.115^*^	0.101	Grade	0.066	0.067
PR × Grade	/	0.132^*^	PA × Grade	/	−0.014
*R* ^2^	0.015	0.032	*R* ^2^	0.223	0.223
Δ*R*^2^	0.013	0.017	Δ*R*^2^	0.004	0.000
F	2.359	3.459^*^	F	44.931^**^	29.890^**^

* p < 0.05,

** p < 0.01.

To comprehensively understand the specific mechanism of grade moderation and the overall model configuration, we conducted further analyses which involving three nested models. Model 1 includes the independent variable (PR). Model 2 adds the moderator variable (grade) to Model 1. Model 3 enhances explanatory power by incorporating the interaction term (PR × Grade) into Model 2. Here, we use the unstandardized coefficient B to report the specific moderating effects of grade. As shown in Table 12, model 3 reveals significant coefficients for the interaction terms “PR × Grade 3.0” (*B* = 0.543, *t* = 2.436, *p* = 0.015^*^) and “PR × Grade 5.0” (*B* = 0.697, *t* = 3.056, *p* = 0.002^**^), demonstrating grade-specific moderation effects on the PR-BI relationship at grades 9 and 11.

**Table 12:** Grade moderation effects (in detail).

**Variables**	**Model 1**	**Model 2**	**Model 3**
Constant	3.409^**^	2.906^**^	2.880^**^
	(56.618)	(17.969)	(17.924)
PR	0.052	0.027	−0.408^*^
	(0.730)	(0.378)	(−2.477)
Grade 1.0 (Reference item)	-	-	-
Grade 2.0		0.507^*^ (2.428)	0.534^*^ (2.578)
Grade 3.0		0.606^**^ (2.849)	0.659^**^ (3.082)
Grade 4.0		0.777^**^ (3.792)	0.804^**^ (3.946)
Grade 5.0		0.248 (1.131)	0.287 (1.317)
Grade 6.0		0.749^**^ (3.071)	0.772^**^ (2.981)
PR × Grade 2.0			0.461 (1.839)
PR × Grade 3.0			0.543^*^ (2.436)
PR × Grade 4.0			0.430 (1.777)
PR × Grade 5.0			0.697^**^ (3.056)
PR × Grade 6.0			0.444 (1.514)
*R* ^2^	0.002	0.061	0.092
F	*F =* 0.532	*F =* 3.380	*F =* 2.809
	*p* = 0.466	*p* = 0.003	*p* = 0.002
Δ*R*^2^	0.002	0.060	0.031
ΔF	*F =* 0.532	*F =* 3.944	*F =* 2.055
	*p* = 0.466	*p* = 0.002	*p* = 0.071

The dependent variable is BI;

* *p* < 0.05,

** *p* < 0.01; The values in parentheses are *t*-values.

## 6 Discussion

The primary objective of this study was to investigate key determinants influencing rural secondary school students' adoption of AI chatbots for mental health education support. This discussion juxtaposes empirical findings with research hypotheses to elucidate mechanisms underlying behavioral intention toward AI chatbot utilization.

### 6.1 Performance expectancy, effort expectancy, social influence and behavioral intention

As hypothesized, PE, EE, and SI demonstrated significant positive correlations with BI. Consistent with prior research, PE emerged as the strongest predictor of technology adoption (109). This suggests rural adolescents' conviction in AI chatbots' efficacy for mental health improvement constitutes a pivotal adoption driver.

As AI chatbots are digital devices, their ease of use strongly impacts users' BI to adopt and continue usage (110). While general technological proficiency with digital devices among Chinese students is relatively high (111), rural students' limited access to AI technologies creates unique usability challenges. Overly complex interaction mechanisms may result in user disengagement and eventual discontinuation of use. Furthermore, inadequate usability design of the chatbots may hinder teachers' capacity to deliver essential technical assistance.

According to the findings, the more rural secondary school students perceived social support for chatbot use, the higher their BI. Peer influence, teacher support, and parental input are critical in rural settings. Therefore, AI chatbots should be embedded in rural educational practices by creating a supportive community in rural school settings and encouraging students to use the devices.

### 6.2 Perceived anthropomorphism and behavioral intention

Moreover, anthropomorphic cues—such as facial expressions, human-like voice tone, and emotionally resonant responses—can enhance adolescents' emotional engagement with chatbots by triggering social cognitive mechanisms similar to those activated in human–human interaction (112). These features reduce perceived psychological distance and promote trust by making the AI appear more intentional and empathetic (113). In therapeutic contexts, trust is crucial for fostering self-disclosure and sustained use (114–116). When adolescents feel the chatbot “understands” them emotionally, they are more likely to share private thoughts and continue using the tool for support—especially in environments where human contact is limited or stigmatized.

### 6.3 Perceived risk and behavioral intention

PR exhibited a negative association with BI, where privacy concerns significantly deterred adoption. This aligns with existing evidence (117). From a neuropsychological perspective, perceived risk may disrupt adolescents' emotional development by overstimulating brain regions associated with threat processing (118). These areas, when hyperactivated by digital threats like privacy breaches, impair emotion regulation control systems (119). This imbalance can heighten anxiety, reduce trust, and lead to withdrawal from AI-assisted tools intended for mental health support (120–122), thereby limiting their therapeutic effectiveness among rural adolescent.

### 6.4 Hedonic motivation and behavioral intention

Contrary to our hypotheses, HM demonstrated no significant correlation with BI. This suggests rural adolescents do not prioritize recreational features as critical adoption determinants—a finding contradicting prior investigations (123, 124). A potential explanation is that, as Digital Natives (125), students maintain frequent exposure to digital technologies and demonstrate a high level of interest in AI systems (126), which may shape their expectations of smart systems' inherent hedonic properties. Concurrently, instrumental utility may outweigh recreational value, with students prioritizing practical therapeutic assistance over entertainment features. However, this does not imply that hedonic design should be neglected. Rather, AI chatbots should incorporate basic entertainment functionalities commonly found in electronic products, while emphasizing practical effectiveness.

### 6.5 The moderating effects of gender and grade

First, we examine the moderating factors affecting the relationship between PR and BI. Our study found that gender did not significantly moderate the relationship between PR and BI. Owing to diminishing gender stereotyping and the structural transition of Chinese rural communities to “semi-familiarity societies” (127–130), women's need for rigorous privacy management as a strategy to maintain social image has decreased. Furthermore, digital-native adolescents exhibit technology usage patterns characterized by diminished gender disparities, resulting in comparable technology acceptance behaviors across genders among rural students. The moderating effect of grade level can be ascribed to the structural pressures that students in grade 9 and grade 11 are confronted with. At this stage in China's education system, they are at the critical juncture of universal-vocational streaming and subject selection (131, 132). As a result, they are in desperate need of mental health support. Meanwhile, to prevent new pressures caused by privacy violations, the potential risks of AI technology.

Then, Regarding the relationship between PA and BI, rural participants' limited AI exposure indicated stronger functional orientation in technology adoption decisions. PA demonstrated an inverted U-shaped relationship with BI, where excessive anthropomorphism decreased adoption likelihood (133). These patterns showed no significant variation by gender or grade level, confirming the absence of moderation effects.

### 6.6 Suggestions

Based on the aforementioned findings, it is evident that multiple factors must be considered when developing a mental health chatbot for rural middle school students.

First, it is essential to enhance the technical performance of the chatbot. It is necessary to ensure that the algorithm can accurately identify early signs of mental health disorders (e.g., depression, anxiety) and provide targeted interventions such as relaxation training and time management. Concurrently, real-time monitoring of psychological states and adaptive feedback mechanisms are essential. Collaboration between educational authorities and certified mental health institutions should be established to develop teleconsultation modules for clinical-grade support. Next, cartoon-style icons and hierarchically simplified operational workflows aligned with adolescent cognitive patterns are required to reduce usability barriers. Supplementary graphic manuals and instructional videos must be provided to facilitate technical proficiency among teachers and students. To expand social influence, schools and education departments should promote chatbot adoption through teacher training workshops and parental awareness programs, thereby improving credibility and acceptance in educational settings. A home-school coordination platform is recommended to enable real-time parental access to students' psychological data for early intervention. Anthropomorphic features such as simulated gestures and emotional language processing can significantly strengthen user emotional bonds and retention rates (134). Therefore, natural language processing should prioritize colloquial expressions over technical jargon to align with adolescents' linguistic habits. Given rural students' dialect preferences (135), dialect recognition modules must be integrated to ensure interaction accuracy. Dynamic effects mimicking human behaviors like nodding and blinking can further enhance user immersion. Robust data protection mechanisms are critical. During initial onboarding, clearly communicate the scope of data collection, usage protocols, and encryption procedures to users. End-to-end encryption technology should be employed to secure chat logs and personal information during transmission and storage. Additionally, anonymization options are necessary to alleviate stigma-related concerns. Finally, implement personalized content delivery systems. Leveraging machine learning algorithms, tailor interventions for subgroups with distinct traits (e.g., high academic stress, introversion), thereby addressing diverse needs and improving user trust and engagement.

## 7 Research limitations and future directions

This study explores the emerging field of AI chatbot applications in mental health education for rural secondary school students. To better align with the specific conditions of the educational context, two external variables, perceived risk (PR) and perceived anthropomorphism (PA), have been integrated based on the UTAUT2 model. Valid data were obtained through standardized questionnaires distributed to students in rural secondary schools in Zhoukou, China, and a structural equation model was developed for validation. Findings revealed that PE, EE, SI, and PA were positively correlated with BI, while PR showed a significant negative correlation. HM did not reach statistical significance. Additionally, gender and grade exhibited significant moderating effects. Practical recommendations include optimizing chatbot functionality, streamlining operational workflows, amplifying social influence, and enhancing user experience.

However, this study has limitations. First, the sample was geographically concentrated in Zhoukou City, a single rural area in central China. This site was chosen due to its large rural population and representative educational characteristics, which broadly reflect the conditions of many rural regions in China. While this provides valuable insights, it may not fully capture the diversity of rural areas nationwide in terms of culture, infrastructure, and digital access. Therefore, future research should consider more geographically and demographically diverse sampling to enhance external validity and improve the generalizability of the findings. Second, the questionnaire-based quantitative methodology, while generating robust empirical data, failed to capture qualitative dimensions of students' psychological experiences. In response, subsequent studies could adopt mixed-methods approaches such as in-depth interviews and ethnographic observation. These methods will allow researchers to gain deeper insights into students' unmet needs and emotional dynamics. Furthermore, a limitation of the present study is the absence of qualitative interviews, which could have offered more nuanced insights into participants' experiences with the AI chatbot. Future research should consider incorporating such qualitative methods, alongside comparisons with traditional mental health education, to gain a deeper understanding of user perceptions and preferences.

## Data Availability

The raw data supporting the conclusions of this article will be made available by the authors, without undue reservation.
